# Evaluation on displacement risks of dismantling temporary lining in tunnel and optimization on temporary lining configuration

**DOI:** 10.1038/s41598-023-35047-1

**Published:** 2023-05-16

**Authors:** Huijian Zhang, Kai Liu, Pan Cao, Gongning Liu

**Affiliations:** 1 School of Civil Engineering, Key Laboratory of Transportation Tunnel Engineering, Ministry of Education, Southwest Jiaotong University, No. 111, North Section, Second Ring Road, Jinniu District, Chengdu, 610031 Sichuan China; 2 Department of Civil and Environmental Engineering, Louisiana State University, Baton Rouge, LA USA

**Keywords:** Civil engineering, Mechanical engineering

## Abstract

In tunnel engineering, the temporary lining is adopted as an effective countermeasure in mitigating tunnel failure potential, often featured by extra-large cross-sections and/or driven through weak ground conditions. However, dismantling temporary linings negatively impacts primary linings. In this paper, the comprehensive research is conducted on the displacement risk caused by dismantling temporary lining based on two alternative tunneling methods (TM-1 and TM-2). Besides, the following three influence factors are taken into consideration: the axial forces in temporary linings, the thickness of preliminary linings, and the deformation modulus of ground. After that, the tunneling method optimization plan is proposed from the view of these three influence factors. The results show that TM-1 always induces invert uplift, whereas TM-2 mainly brings about invert uplift or sidewall bulging depending on which transverse or vertical linings are dominant in terms of axial force values. For TM-2, the axial force in transverse linings can suppress the development of maximum deformation increment (MDI) value at invert when the axial forces in transverse linings are smaller than those in vertical linings. It is also found that with the further increase of the axial force in transverse linings in TM-2, MDI relocates to the sidewall. Moreover, on the basis of the displacement risk evaluations, an optimization on the temporary lining configurations has been developed by replacing temporary linings with pre-tension anchor cables to reduce the risk of dismantling temporary linings. All the research results can provide some important reference for the similar tunnel engineering in the future.

## Introduction

Nowadays, a tunnel is designed and constructed towards large span and section to accommodate various needs^1–7^, and the lining is vital to the tunnel stability during construction^8–10^. When excavating a large-section tunnel in a weak stratum^11^, the temporary lining is usually employed to reduce the span with the help of various sequential excavation methods, which include the center diaphragm method^12^, the center cross diaphragm method^13,14^, the side drift method^15,16^, and other tunneling methods^17–20^. Yiouta-Mitra et al*.*^21^ used a sensitivity analysis method to assess the impact of various geotechnical parameters on the internal forces and displacements of temporary tunnel lining, and obtained the optimal parameters. Admittedly, the temporary lining stabilizes the ground and controls displacement during excavation. However, dismantling temporary linings often negatively affects primary linings because the loads sustained by temporary linings would be transferred to the surrounding bearing elements^22^.

Traditionally, controlling the dismantling length of the temporary lining per round is often used to mitigate these adverse effects. Zhang et al.^23,24^ pointed out that the risk of dismantling temporary tunnel lining can be reduced, and the structure’s safety can be ensured by using step-by-step dismantling temporary lining and installation of the secondary lining. The previous studies^25–29^ recommended the dismantling length per round using 6 ~ 12 m. Zhang et al*.*^30^ pointed out that with the increase of the one-time demolition length of temporary lining, the safety factor of tunnel would first increase and then decrease, and it was suggested that the reasonable one-time demolition length of the temporary lining should be 9 m. Wang et al*.*^31^ optimized the demolition length of the temporary lining (from 6 m to 12 m) and the amount of excavation section (from 7 excavation sections to 4 excavation sections) of the super-large section subway tunnel, which significantly shortened the construction period and cost. Zhang et al*.*^32^ pointed out that the timing of dismantling of vertical temporary linings is the main factor affecting tunnel safety, and it is in the best interest of tunnel safety to dismantle the vertical temporary linings at last. However, it has to be pointed out that this suggested dismantling length is too short to carry out the subsequent parallel installation of the final linings and therefore will seriously affect the tunneling efficiency. From the above, it is essential to analyze, without considering the three-dimensional effect, the deformation increments about the preliminary lining led by dismantling the temporary linings and optimizing the tunneling method to reduce the adverse effects.

**Figure 1:**
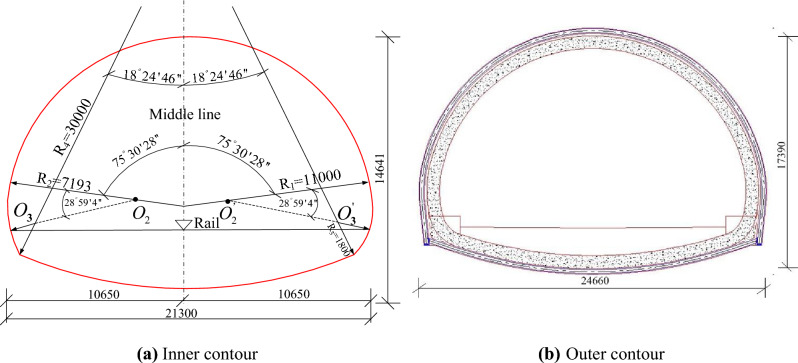
Tunnel profile of the four-line railway tunnel.

**Figure 2:**
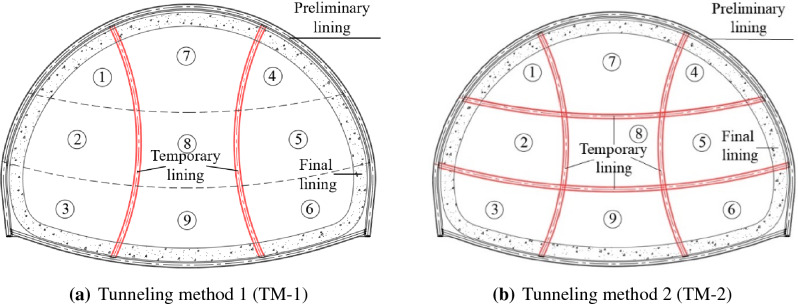
Two alternative tunneling methods.

The previous works done by our research group (Zhang et al.^33^) focused on the displacement increment led by the dismantling of temporary lining before the preliminary lining closed (none closed-loop preliminary lining loading). However, there is no further research on the risk assessment led by the dismantling of temporary lining after the preliminary lining closed (closed-loop preliminary lining loading). This study is based on the same four-line railway station tunnel in Zhang et al.^33^, and the cross-section profile is shown in Fig. 1. Two alternative tunneling methods proposed in the tunneling methods (plans) comparison and optimization stage, marked as tunneling method 1 (abbreviated as TM-1) and tunneling method 2 (abbreviated as TM-2) respectively, are presented in Fig. 2. The corresponding lining parameters are summarized in Table 1. In this paper, the comprehensive research is conducted on the displacement risk caused by dismantling temporary lining based on these two alternative methods. Besides, the following three influence factors are taken into consideration: the axial forces in temporary linings, the thickness of preliminary linings, and the deformation modulus of ground. After that, in order to reduce the risk of dismantling temporary linings, tunneling method optimization plan is proposed. All the research results can provide some important reference for the similar tunnel engineering in the future.

**Table 1:** Details of the preliminary lining, temporary lining and final lining.

Preliminary lining				Temporary lining				Final lining	
Steel ribs		Shotcrete		Steel ribs		Shotcrete		Reinforced concrete	
Size	Spacing	Grade	Thickness	Size	Spacing	Grade	Thickness	Grade	Thickness
I-28b	60 cm	C25	30 cm	I-25b	60 cm	C25	20 cm	C35	90 cm

## Calculation model

The bedded-beam model is widely used in tunnel analyses and designs, as suggested by the International Tunnel Association Tunnel Design Working Group^34^, and in research relevant to tunnel engineering^35,36^, which is adopted in this paper.

In the numerical simulation, the tunnel section is built according to the tunnel profile in Fig. 1. The preliminary lining, as well as temporary lining, is modeled using the *beam* structure element (Beam 3), and the interaction between the preliminary lining and ground is realized by spring (Link 10). Note that the interaction springs around the tunnel perimeter can only bear compressive loads, and the forces of springs will be set to 0 when tension appears. The outer ends of the springs are fixed. The calibration of the bedded-beam model can be referred to the previous works conducted by our research group (namely, Zhang et al.^33^). The two tunneling methods involved in this study are two alternative methods based on the same background project mentioned in Zhang et al.^33^.

**Figure 3:**
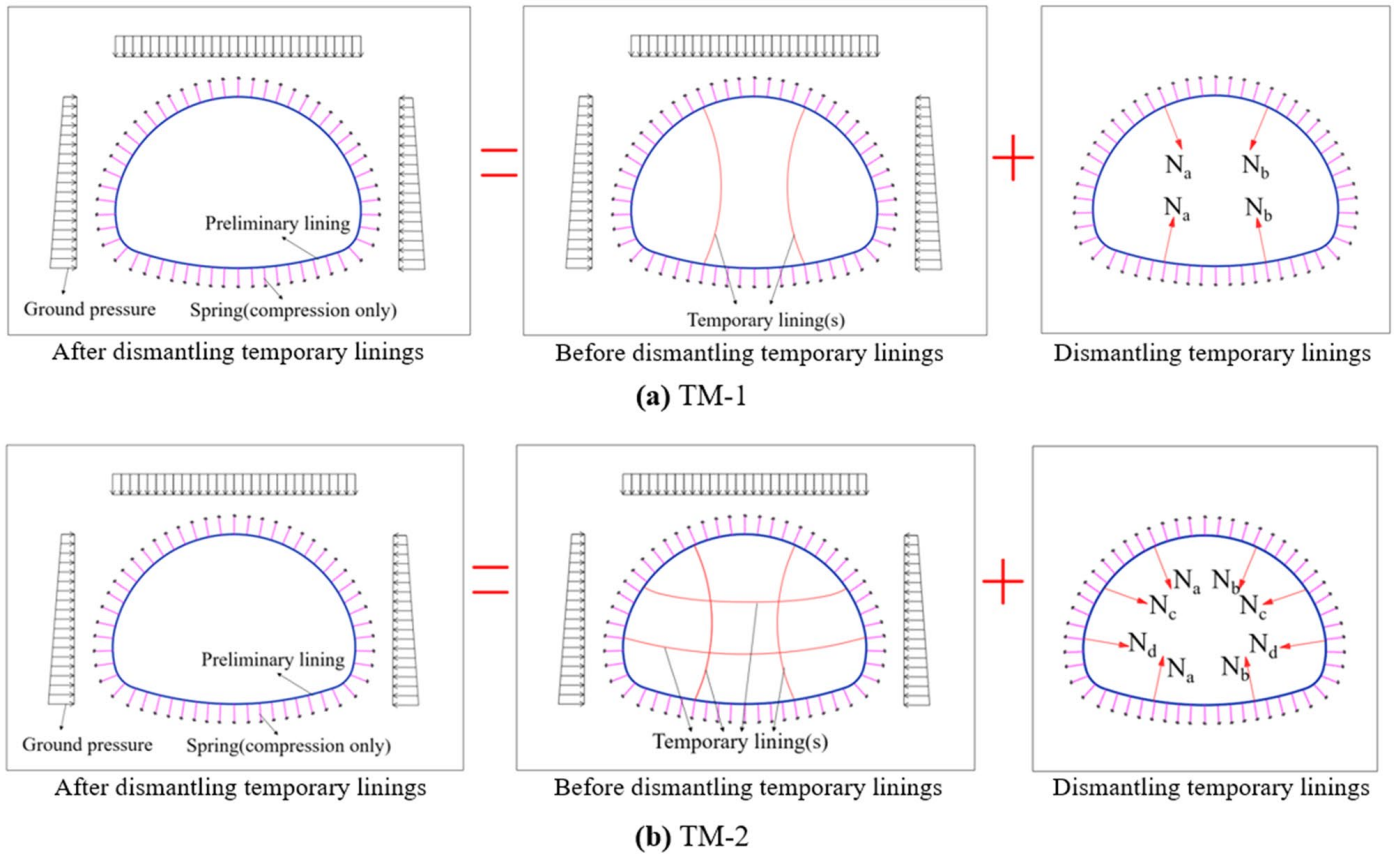
Calculation models of dismantling the temporary linings by TM-1 and TM-2.

**Figure 4:**
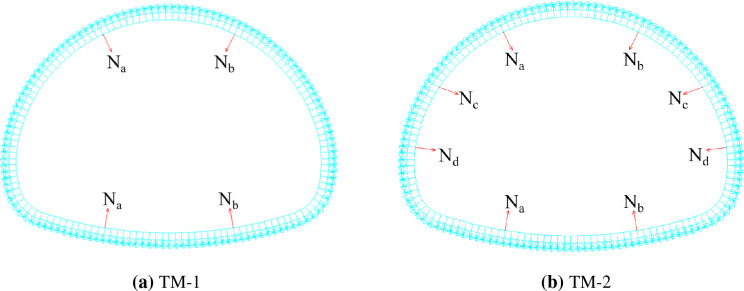
Numerical calculation models of TM-1 and TM-2.

From the perspective of mechanical equilibrium, removing the temporary lining will transfer to the surrounding bearing elements the loads that the temporary lining sustains (see Fig. 3). Hence, the numerical models of TM-1 and TM-2 are established, respectively, as shown in Fig. 4a,b. It can be seen from the numerical models that there are three influence factors, i.e. the axial forces in the temporary lining (*AFTL*), the stiffness of preliminary linings (referred to the thickness of preliminary lining in this paper, abbreviated as TPL), and the deformation modulus about ground (*DMG*).

For the magnitude of the parameters entered in the analysis, different calculation cases are assumed based on the on-site monitoring data as well as suggestions from the literatures. With regard to AFTL, the maximum value monitored by the background project is nearly 700 kN/m, while some literature^27,28,37^ points out that the AFTL can be up to 1026 kN/m. Besides, for the TPL, 400 mm is adopted in the background project, while it is suggested greater than 200 mm by the design experiences and guidelines. For the DMG, it is a wide range from the bad to the good ground, but it is more meaningful to focus on the sensitive range of 50 Mpa/m ~ 500 Mpa/m. Therefore, in the following numerical simulation, it is reasonable to set the AFTL in the range of 0 ~ 800 kN/m, the TPL between 200 mm and 700 mm, and the DMG from 50 Mpa/m to 500 Mpa/m.

### Calculation results based on tunneling method 1 (TM-1)

**Figure 5:**
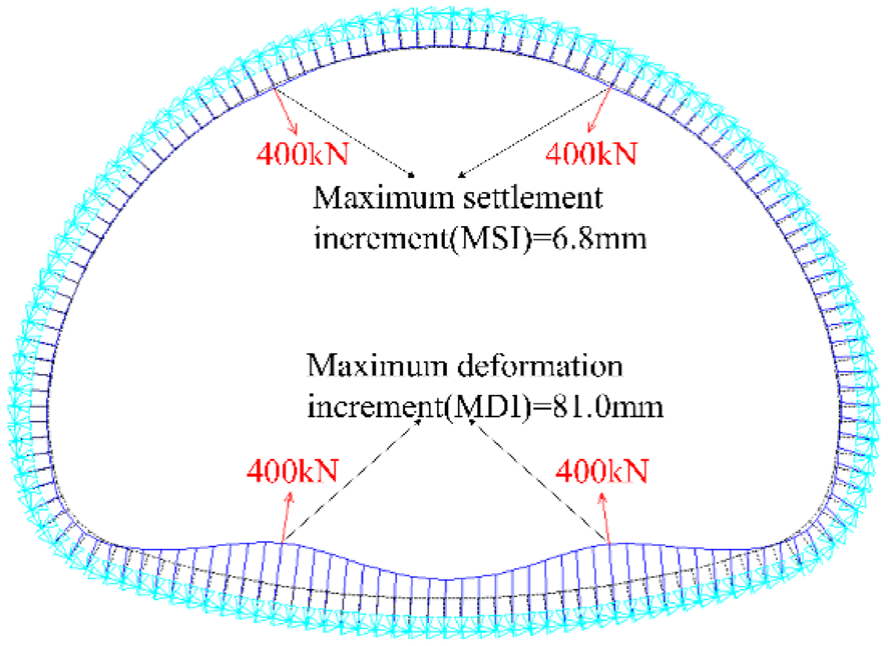
Deformation increment profile of preliminary linings led by the dismantling of temporary linings based on TM-1.

Based on the numerical model in Fig. 4a, the calculation parameters are initially set as follows: the TPL is 200 mm; the *DMG* is 50 MPa/m; and N_a_ and N_b_ are both set as 400 kN/m. The obtained result is presented in Fig. 5. Due to the curvature difference, the deformation increments about preliminary linings led by dismantling temporary linings are mainly shown as invert uplift. The maximum settlement increment (MSI) about the arch is 6.8 mm, while the MDI of the invert of the preliminary lining is 81.0 mm. Subsequently, the MDI of preliminary lining would be employed to quantify the impact of dismantling temporary linings.

### Influence factor—AFTL (N_a_ and N_b_)

The DMG is set as 150 MPa/m, and TPL is set as 400 mm. Two cases are considered to investigate the AFTL (N_a_ and N_b_): keeping N_a_ = N_b_ and varying N_a_ and N_b_ independently.

#### Keeping N_a_ = N_b_

**Figure 6:**
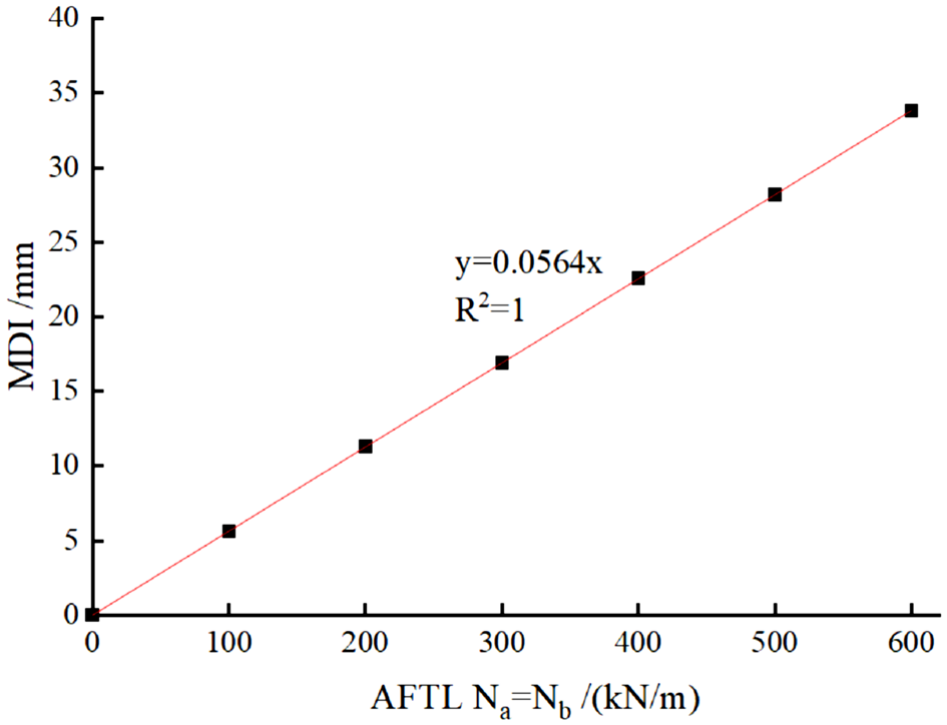
MDI versus AFTL when keeping N_a_ = N_b_ in TM-1.

The force parameters and calculation results are shown in Table 2 and Fig. 6. A linear relationship between N_a_ (or N_b_) and MDI is displayed in the figure. The MDIs increase with *AFTL*. Generally, it is found that MDI increases by 5.6 mm for each 100 kN/m increasement in AFTL.

**Table 2:** Calculation cases and results for N_a_ = N_b_ in TM-1.

Calculation case	MDI of the preliminary lining/mm
N_a_ = N_b_ = 0 kN/m	0
N_a_ = N_b_ = 100 kN/m	5.6
N_a_ = N_b_ = 200 kN/m	11.3
N_a_ = N_b_ = 300 kN/m	16.9
N_a_ = N_b_ = 400 kN/m	22.6
N_a_ = N_b_ = 500 kN/m	28.2
N_a_ = N_b_ = 600 kN/m	33.8

#### Varying N_a_ and N_b_ independently

**Figure 7:**
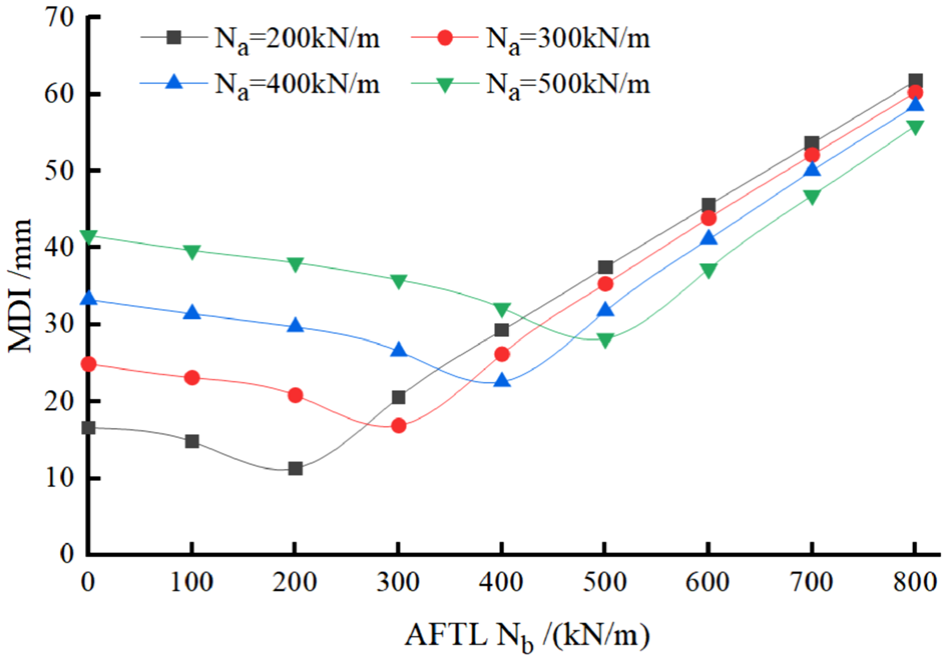
The relationship among the MDI, N_a_, and N_b_ when axial forces N_a_ and N_b_ vary independently in TM-1.

The force parameters and calculation results are shown in Table 3 and Fig. 7. From Fig. 7, the N_b_—MDI distribution curve exhibits nonlinear behavior under a constant N_a_. Specifically, under a constant N_a_, MDI decreases with the increase of N_b_ before N_a_ = N_b_. After that, MDI increases with the further increase of N_b_ (N_b_ > N_a_). Note that the minimum MDI is achieved when N_a_ = N_b_. In other words, the most favorable situation can be achieved when the axial forces in the two vertical temporary linings keep the same N_a_ = N_b_. In addition, the MDI is mainly subject to the dominant axial force when N_a_ and N_b_ are different.

**Table 3:** Calculation cases and results (MDI/mm) for N_a_ and N_b_ independently varying in TM-1.

		N_b_/(kN/m)								
		0	100	200	300	400	500	600	700	800
N_a_/(kN/m)	200	16.6	14.8	11.3	20.6	29.3	37.5	45.6	53.6	61.7
	300	24.9	23.2	20.9	16.9	26.2	35.3	43.9	52.1	60.3
	400	33.3	31.4	29.7	26.6	22.6	31.8	41.1	50.1	58.5
	500	39.7	39.7	38.1	35.8	32.1	28.2	37.3	46.8	55.9

### Influence factor—TPL

**Figure 8:**
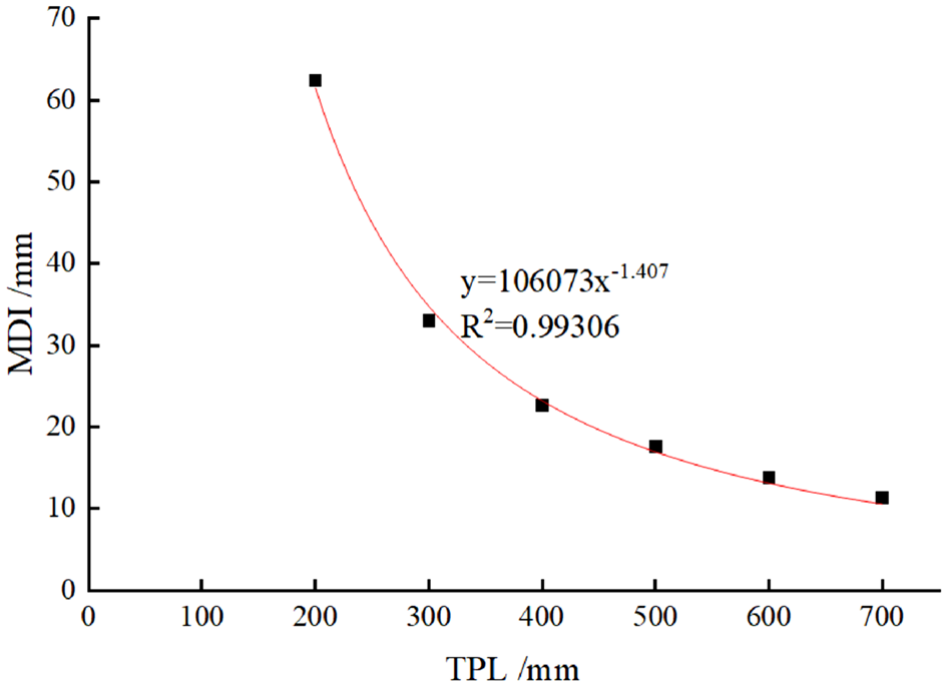
MDI versus the TPL in TM-1.

In this subsection, N_a_ and N_b_ are set as 400 kN/m, and the DMG is set as 150 MPa/m. The TPL changes in the range of 200 ~ 700 mm with an interval of 100 mm. The relationship between MDI and TPL can be captured by a power function (see Fig. 8). It should be pointed out that MDI reduces rapidly when TPL is small; and that with further increase of TPL, MDI decreases slowly. This finding implies that further increasing TPL will not significantly decrease the risks of dismantling temporary linings when the preliminary lining is competent (or TPL is large).

### Influence factor—DMG

**Figure 9:**
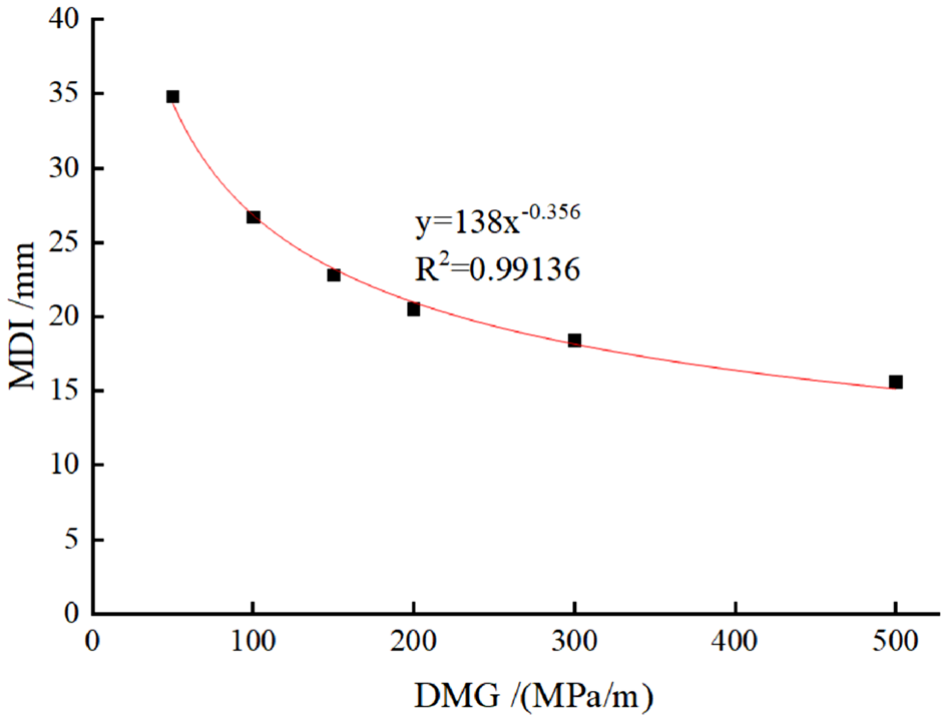
MDI versus the DMG in TM-1.

In this subsection, N_a_ and N_b_ are set as 400 kN/m and TPL is set as 400 mm. The DMG is set to 50 MPa/m, 100 MPa/m, 150 MPa/m, 200 MPa/m, 300 MPa/m, and 500 MPa/m, respectively. The corresponding calculation results are shown in Fig. 9. In Fig. 9; the MDI decreases with the increase of DMG (from soft ground to hard ground), which can also be described by a power function. Similarly, with the increase of DMG, MDI decreases slowly. This finding implies that improving the ground condition can be an effective measure in reducing the risks of dismantling temporary linings only when the ground is weak.

## Discussion

**Figure 10:**
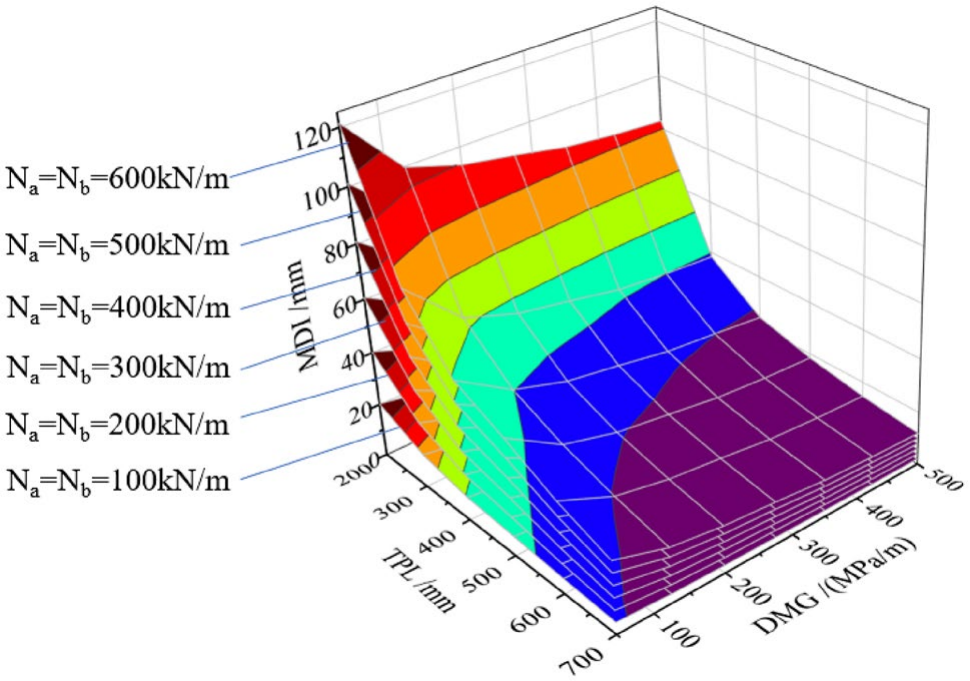
3D surface graphs among the MDI, TPL, DMG and AFTL.

A total of 216 calculation conditions as well as the corresponding result involving all three influence factors are listed in Table 4 and Fig. 10.

**Table 4:** Calculation cases and results (MDI/mm) for involving all the three influence factors in TM-1.

TPL/mm	DMG/(MPa/m)	AFTL (N_a_ = N_b_)/(kN/m)					
		100	200	300	400	500	600
200	50	20.3	40.5	60.8	81.0	101.3	121.5
	100	16.6	33.2	49.9	66.5	83.1	99.7
	150	15.6	31.2	46.8	62.4	78.0	93.6
	200	15.0	30.1	45.1	60.1	75.1	90.2
	300	14.4	28.8	43.2	57.6	72.0	86.4
	500	13.8	27.6	41.5	55.3	69.1	82.9
300	50	12.4	24.7	37.1	49.4	61.8	74.2
	100	9.2	18.4	27.6	36.8	46.0	55.2
	150	8.1	16.2	24.3	32.4	40.5	48.5
	200	7.5	15.0	22.5	30.0	37.5	45.0
	300	6.9	13.7	20.6	27.5	34.4	41.2
	500	6.4	12.8	19.2	25.5	31.9	38.3
400	50	8.7	17.4	26.1	34.8	43.5	52.2
	100	6.6	13.2	19.9	26.5	33.1	39.7
	150	5.6	11.3	16.9	22.6	28.2	33.8
	200	5.1	10.3	15.4	20.5	25.6	30.8
	300	4.6	9.2	13.8	18.4	23.0	27.5
	500	4.0	8.0	11.9	15.9	19.9	23.9
500	50	7.3	14.5	21.8	29.0	36.3	43.5
	100	5.2	10.5	15.7	20.9	26.1	31.4
	150	4.4	8.8	13.2	17.6	21.9	26.3
	200	3.9	7.9	11.8	15.8	19.7	23.6
	300	3.5	6.9	10.4	13.9	17.3	20.8
	500	3.1	6.1	9.2	12.3	15.3	18.4
600	50	5.7	11.3	17.0	22.6	28.3	33.9
	100	4.2	8.4	12.6	16.8	21.1	25.3
	150	3.6	7.1	10.7	14.3	17.9	21.4
	200	3.2	6.4	9.6	12.8	16.0	19.2
	300	2.8	5.6	8.4	11.2	14.0	16.7
	500	2.4	4.9	7.3	9.7	12.2	14.6
700	50	4.5	8.9	13.4	17.8	22.3	26.7
	100	3.4	6.8	10.2	13.6	17.0	20.4
	150	2.9	5.8	8.8	11.7	14.6	17.5
	200	2.6	5.3	7.9	10.5	13.2	15.8
	300	2.3	4.6	6.9	9.2	11.5	13.8
	500	2.0	4.0	6.0	8.0	10.0	12.0

As can be seen from Fig. 10, the peak MDI with 121.5 mm is reached when the AFTL reaches the highest value (i.e. 600 kN/m) and both the TPL and DMG are the lowest (i.e. 200 mm and 50 MPa/m, respectively). The minimum MDI of 2.0 mm is obtained under the opposite condition where the AFTL reaches the lowest value (i.e. 100 kN/m) and both the TPL and DMG are the highest (i.e. 700 mm and 500 MPa/m, respectively). All faces in Fig. 10 show convex downward. The highest gradient of the contour plot is near the peak MDI where the preliminary lining and DMG are weak, indicating that increasing the TPL and improving the DMG can be effective measures in lowering the risks of dismantling the temporary linings when the preliminary lining and ground are weak.

**Figure 11:**
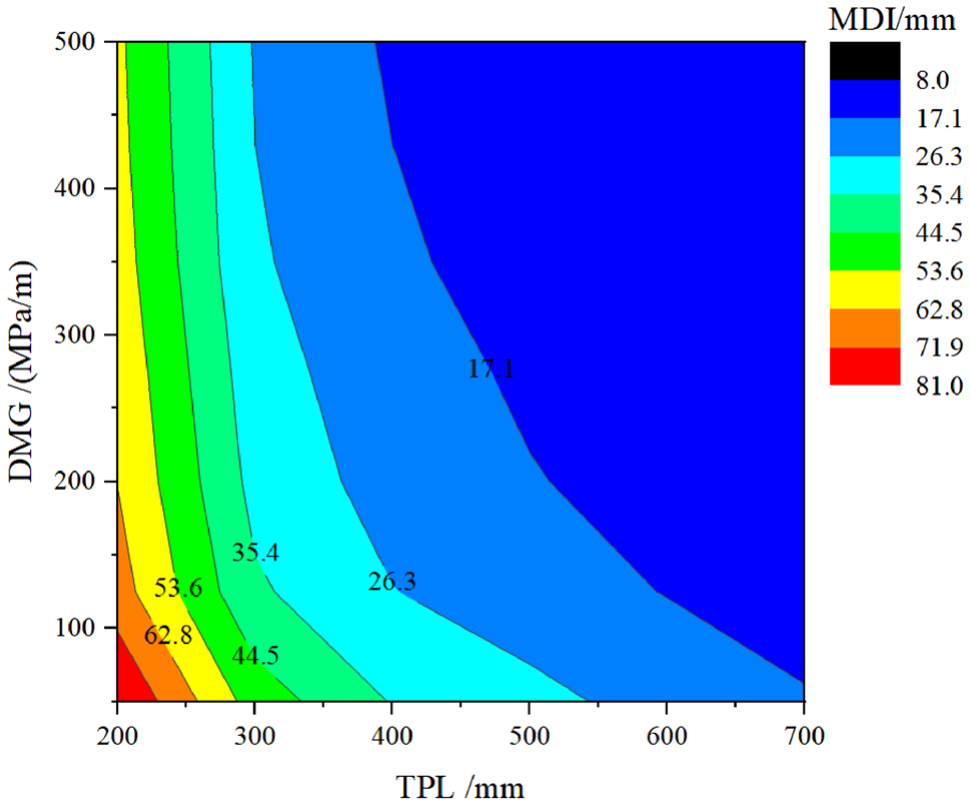
Curve about MDI concerning the TPL and the DMG.

**Figure 12:**
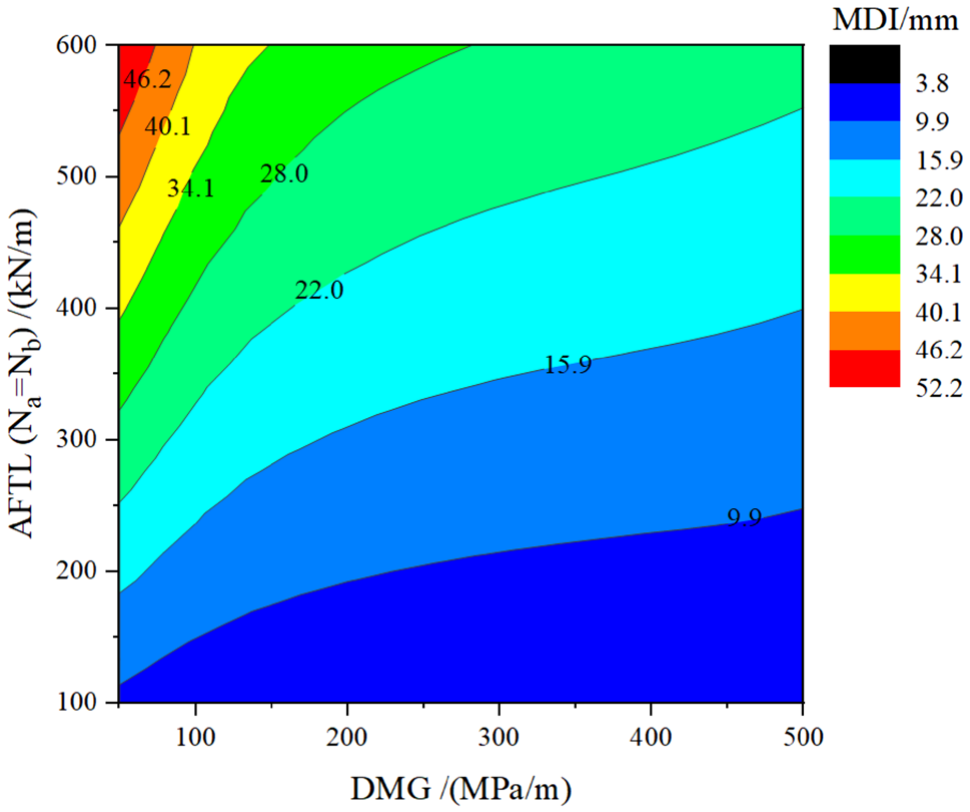
Curve about MDI concerning the DMG and the AFTL.

**Figure 13:**
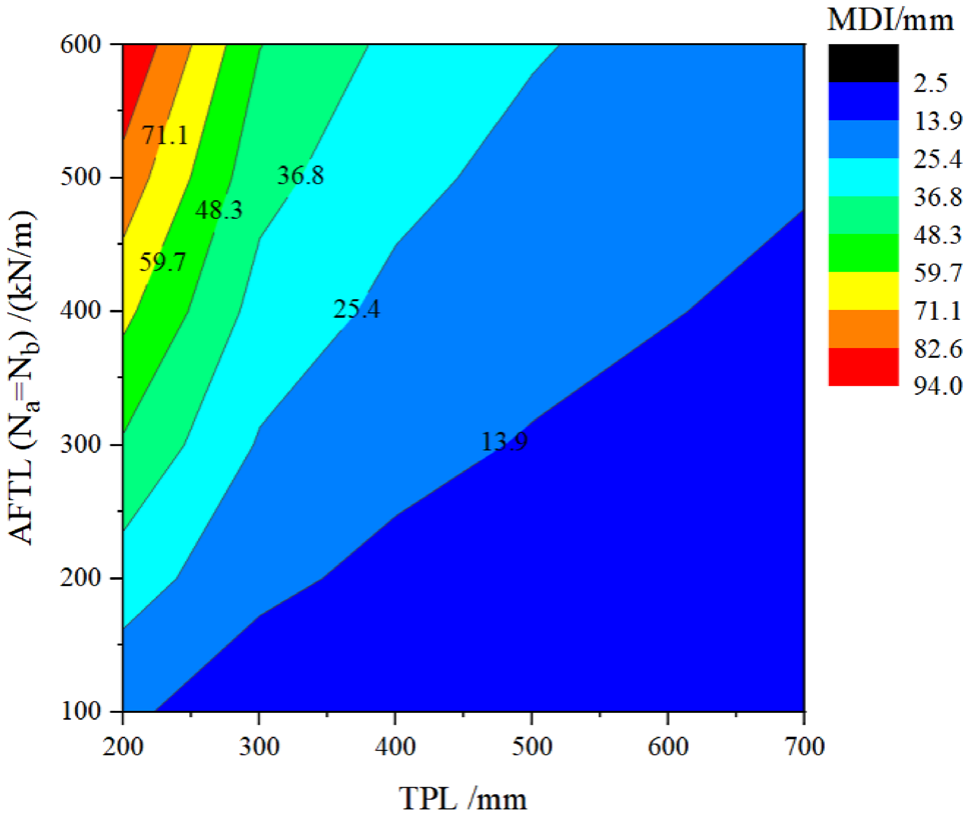
Curve about MDI concerning the TPL and the AFTL.

To better illustrate the impact of the three elements on MDI, three contour plots are extracted from Fig. 10, as shown in Figs. 11, 12 and 13.

Figure 11 presents the influence of the TPL and DMG on MDI. The red section represents the high risks (significant value of the MDI), while the blue section refers to the low risks. The contour lines close to the red area are denser. Along the horizontal axis, increasing the TPL can make the MDI change from the red section to the blue section. Along the vertical axis, increasing the DMG can only make the MDI change from the red section to the yellow section. This indicates that increasing the TPL is more effective in reducing the MDI than increasing the DMG. Increasing the TPL and DMG simultaneously can effectively control the risks of removing the temporary linings.

Figure 12 shows the influence of the AFTL and DMG on the MDI. Along the vertical axis, decreasing the AFTL can make the MDI change from the red section to the blue section. Along the horizontal axis, increasing the DMG cannot make the MDI change from red to blue. This indicates that increasing the AFTL is more effective in reducing the MDI than increasing the DMG. When the axial force N_a_ = N_b_ = 100 kN/m, the risks of dismantling temporary linings are small (the MDI is in blue), and therefore the impact of DMG on the MDI can be ignored. Note that the entire horizontal axis is blue in color.

Figure 13 displays the influence of the *AFTL* and TPL on the MDI. Along the horizontal and vertical axes, increasing the TPL or decreasing the AFTL can make the MDI change from the red section to the blue section. Countermeasures of increasing the TPL and decreasing the *AFTL* simultaneously are effective. Meanwhile, it is noted that when the axial force is small, such as N_a_ = N_b_ = 100 kN/m, the influence of the TPL on the MDI is slight.

### Calculation results based on tunneling method 2 (TM-2)

In TM-2, two more transverse temporary linings are added compared with TM-1. According to Fig. 4b, the AFTLs are marked as N_a_, N_b_, N_c,_ and N_d_. There are still the same three influence factors: *AFTL*, TPL, and DMG. Since the AFTL in TM-2 is more complicated, calculation cases are divided into three groups in the following analyses: keeping N_a_ = N_b_ = N_c_ = N_d_, keeping N_a_ = N_b_ and N_c_ = N_d_, keeping N_a_ = N_b_ and fixing N_c_ = 400 kN/m with varying N_d_, named as Group A, Group B, and Group C, respectively.

### Group A – keeping N_a_ = N_b_ = N_c_ = N_d_

**Figure 14:**
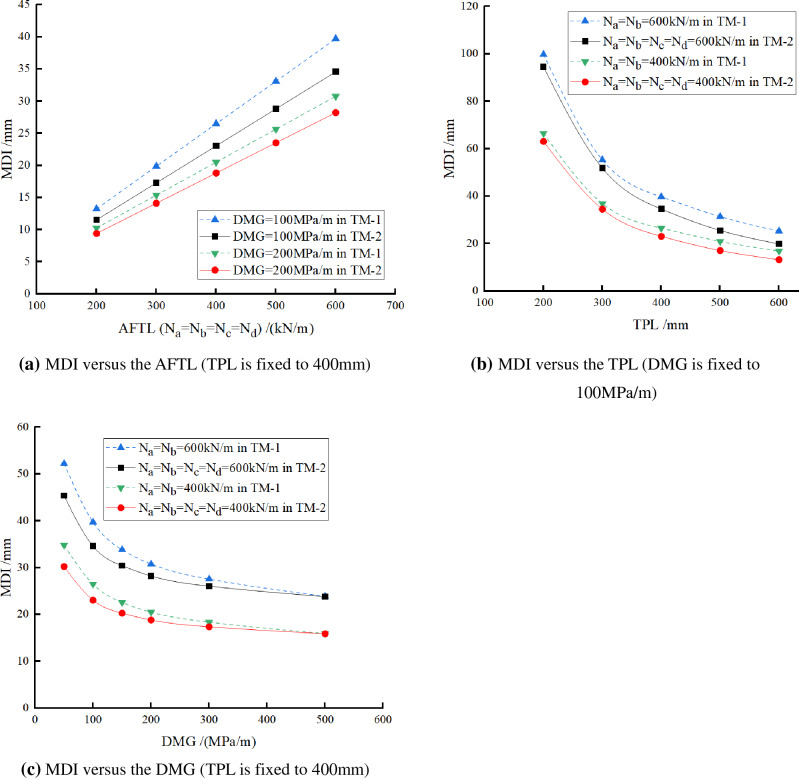
Calculation results of TM-2 (N_a_ = N_b_ = N_c_ = N_d_) and the comparison with TM-1 (N_a_ = N_b_).

It is assumed that the axial forces in the transverse-vertical temporary linings are the same, that is, N_a_ = N_b_ = N_c_ = N_d_. The corresponding calculation cases and results, as well as the comparisons with TM-1 (only vertical temporary linings), are shown in Table 5 and Fig. 14.

1. From the data of each row in Table 5, when the *AFTL* increases from 200 to 400 kN/m or from 300 to 600 kN/m in TM-2, the MDI just doubled. In other words, the MDI is linearly related to the *AFTL* when N_a_, N_b_, N_c,_ and N_d_ increase simultaneously, as shown in Fig. 14a. However, the values of the MDI in TM-2 are smaller than in TM-1. It implies that dismantling the temporary transverse linings can suppress the MDI led by dismantling the vertical temporary linings. Moreover, when TPL and DMG are fixed, the ratio of MDI in TM-2 to that in TM-1 is the same under the same magnitude of axial force.
2. As shown in Fig. 14b, when N_a_, N_b_, N_c,_ and N_d_ are fixed to the same value, the MDI decreases with the TPL and shows a power function relationship. That is consistent with the findings from TM-1. The discrepancy between the solid and dashed curves under the same axial forces increases with the TPL, for example. With the DMG equal to 200 MPa/m, the ratios of the MDIs in TM-2 to those in TM-1 are 0.96, 0.94, 0.92, 0.85, and 0.81 respectively, corresponding to the TPL are 200 mm, 300 mm, 400 mm, 500 mm, and 600 mm. It implies that although the MDI decreases with the TPL, the difference in the MDI between the two tunneling methods increases.
3. As shown in Fig. 14c, when N_a_, N_b_, N_c,_ and N_d_ are fixed to the same value, the MDI reduces with DMG and shows a power function relationship. That is consistent with the findings from TM-1. The differences between the solid and dashed curves under the same axial forces decrease with the DMG before they converge at the same point. It indicates that the absolute values of MDI and the differences in MDI between the two tunneling methods decrease with the DMG.

**Table 5:** Calculation cases and results (MDI/mm) for N_a_ = N_b_ = N_c_ = N_d_ in TM-2 and comparison with TM-1.

TPL/mm	DMG/(MPa/m)	N_a_ = N_b_ = N_c_ = N_d_ = /(kN/m), TM-2				N_a_ = N_b_ = /(kN/m), TM-1				The ratio of (TM-2/TM-1)		
		200	300	400	600	200	300	400	600	200	400	600
200	50	37.50	56.24	74.99	112.49	40.51	60.77	81.02	121.54	0.93	0.93	0.93
	100	31.52	47.28	63.04	94.55	33.24	49.86	66.48	99.73	0.95	0.95	0.95
	150	29.72	44.57	59.43	89.15	31.20	46.80	62.40	93.60	0.95	0.95	0.95
	200	28.71	43.06	57.42	86.12	30.05	45.08	60.10	90.16	0.96	0.96	0.96
	300	27.61	41.42	55.22	82.83	28.80	43.21	57.61	86.41	0.96	0.96	0.96
	500	26.63	39.94	53.26	79.88	27.65	41.47	55.30	82.94	0.96	0.96	0.96
300	50	22.36	33.53	44.71	67.07	24.72	37.08	49.44	74.16	0.90	0.90	0.90
	100	17.25	25.88	34.50	51.75	18.41	27.62	36.83	55.24	0.94	0.94	0.94
	150	15.18	22.77	30.36	45.55	16.18	24.27	32.36	48.54	0.94	0.94	0.94
	200	14.08	21.11	28.15	42.23	14.98	22.48	29.97	44.95	0.94	0.94	0.94
	300	12.97	19.45	25.94	38.90	13.75	20.62	27.50	41.24	0.94	0.94	0.94
	500	12.08	18.12	24.16	36.23	12.77	19.16	25.54	38.31	0.95	0.95	0.95
400	50	15.12	22.68	30.24	45.36	17.40	26.10	34.80	52.20	0.87	0.87	0.87
	100	11.52	17.28	23.04	34.56	13.24	19.86	26.48	39.71	0.87	0.87	0.87
	150	10.14	15.21	20.28	30.41	11.28	16.92	22.56	33.84	0.90	0.90	0.90
	200	9.41	14.11	18.81	28.22	10.25	15.38	20.50	30.76	0.92	0.92	0.92
	300	8.68	13.02	17.36	26.04	9.18	13.77	18.36	27.55	0.95	0.95	0.95
	500	7.93	11.90	15.87	23.80	7.96	11.93	15.91	23.87	1.00	1.00	1.00
500	50	11.22	16.83	22.44	33.65	14.51	21.77	29.02	43.54	0.77	0.77	0.77
	100	8.52	12.77	17.03	25.55	10.46	15.69	20.92	31.37	0.81	0.81	0.81
	150	7.36	11.03	14.71	22.07	8.78	13.16	17.55	26.33	0.84	0.84	0.84
	200	6.71	10.07	13.43	20.14	7.88	11.82	15.76	23.63	0.85	0.85	0.85
	300	6.06	9.09	12.12	18.18	6.93	10.40	13.87	20.80	0.87	0.87	0.87
	500	5.51	8.26	11.01	16.52	6.14	9.21	12.28	18.41	0.90	0.90	0.90
600	50	7.87	11.81	15.74	23.61	11.30	16.95	22.60	33.91	0.70	0.70	0.70
	100	6.61	9.91	13.21	19.82	8.42	12.64	16.85	25.27	0.79	0.79	0.79
	150	5.76	8.64	11.52	17.28	7.15	10.72	14.30	21.44	0.81	0.81	0.81
	200	5.22	7.83	10.44	15.67	6.41	9.61	12.82	19.22	0.81	0.81	0.81
	300	4.64	6.97	9.29	13.93	5.58	8.37	11.16	16.75	0.83	0.83	0.83
	500	4.15	6.23	8.31	12.46	4.86	7.29	9.72	14.58	0.85	0.85	0.85

### Group B – keeping N_a_ = N_b_ and N_c_ = N_d_

**Figure 15:**
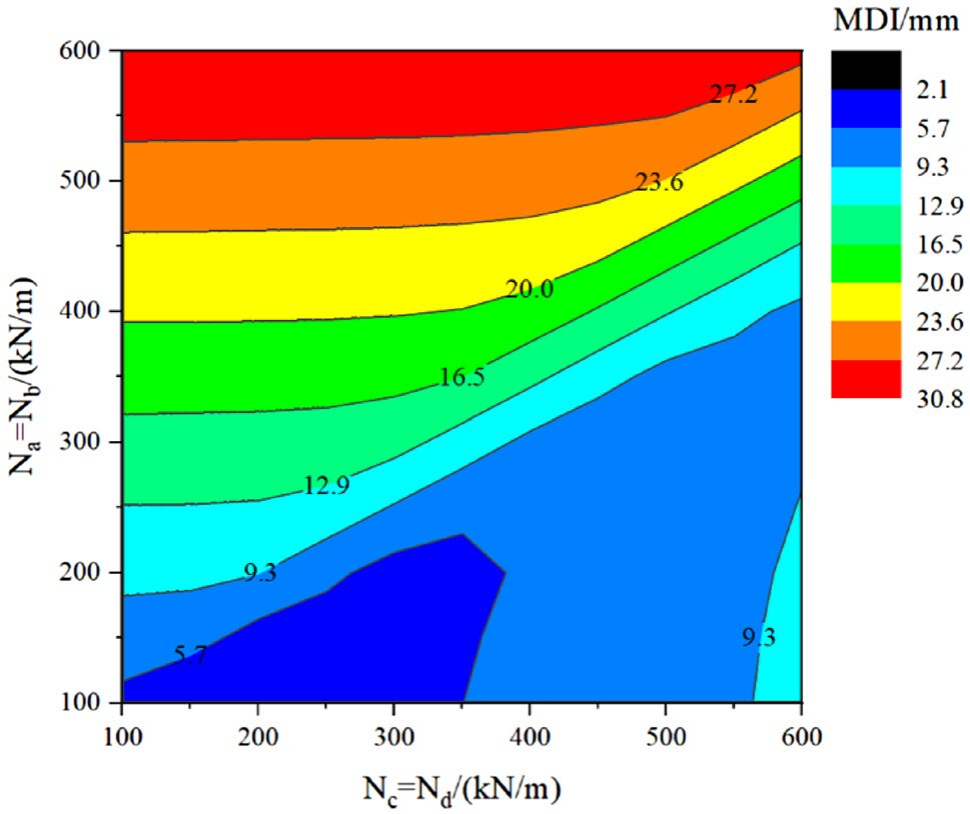
Contour chart about MDI concerning the vertical forces (N_a_ = N_b_) and the transverse forces (N_c_ = N_d_).

This subsection assumes that the axial force in the two transverse temporary linings varies simultaneously (N_c_ = N_d_), as well as those in the two vertical temporary linings (N_a_ = N_b_). However, the axial force in the transverse and vertical temporary lining varies independently. The calculation result is displayed in Table 6 and Fig. 15 under the following conditions: the DMG is set as 200 MPa/m, and TPL is set as 400 mm.

**Table 6:** MDI location under different AFTL in group B.

N_a_ = N_b_ =	N_c_ = N_d_ =			
	200 kN/m	300 kN/m	500 kN/m	600 kN/m
200 kN/m				
400 kN/m

It should be noted first that the MDI location may change when axial force in the transverse temporary lining is more significant than that in the temporary vertical lining, compared with TM-1. Table 6 summarizes the MDI results and locations. Please assume that the axial force in the temporary vertical lining is small (N_a_ = N_b_ = 200 kN/m) to explain the evolution of MDI and its location. When the axial force in the transverse temporary lining (N_c_ and N_d_) increases from 200 to 300 kN/m, the MDI location is still at the invert, but the value of MDI decreases. It means that the MDI and its location are mainly dominated by the vertical forces N_a_ and N_b_, whereas the transverse forces N_c_ and N_d_ only have a suppression effect on the MDI. When N_c_ and N_d_ continue to increase from 300 to 500 kN/m, the MDI location changes from the invert to the sidewall. At this stage, it means the MDI is mainly caused by the transverse forces N_c_ and N_d_. When N_c_ and N_d_ change from 500 to 600 kN/m, the MDI location is still at the sidewall and the value of MDI increases. With the vertical forces N_a_ = N_b_ = 400 kN/m and the transverse forces N_c_ and N_d_ varying from 200 to 600 kN/m, the MDI location is always at the invert, but the deformation increment at the sidewall becomes from low to high progressively. In addition, the value of MDI continuously decreases due to the suppression effect by N_c_ and N_d_.

With the above analysis, understanding Fig. 15 would be quite straightforward. When the vertical forces are large, for example, N_a_ = N_b_ = 400 kN/m, the transverse forces (N_c_ and N_d_) make the MDI decrease when it changes from 100 to 600 kN/m, and the decreasing rate of MDI increases. When N_a_ and N_b_ are small (i.e. 200 kN/m), the MDI will go through light blue—blue—light blue areas with N_c_ and N_d_ evolving from 100 to 600 kN/m. This means that N_c_ and N_d_ suppress the MDI when the MDI locates at the invert (from light blue to blue area), but N_c_ and N_d_ help increase the MDI after the MDI location moves to the sidewall (from blue to light blue area).

### Group C – keeping N_a_ = N_b_ and fixing N_c_ = 400 kN/m

**Figure 16:**
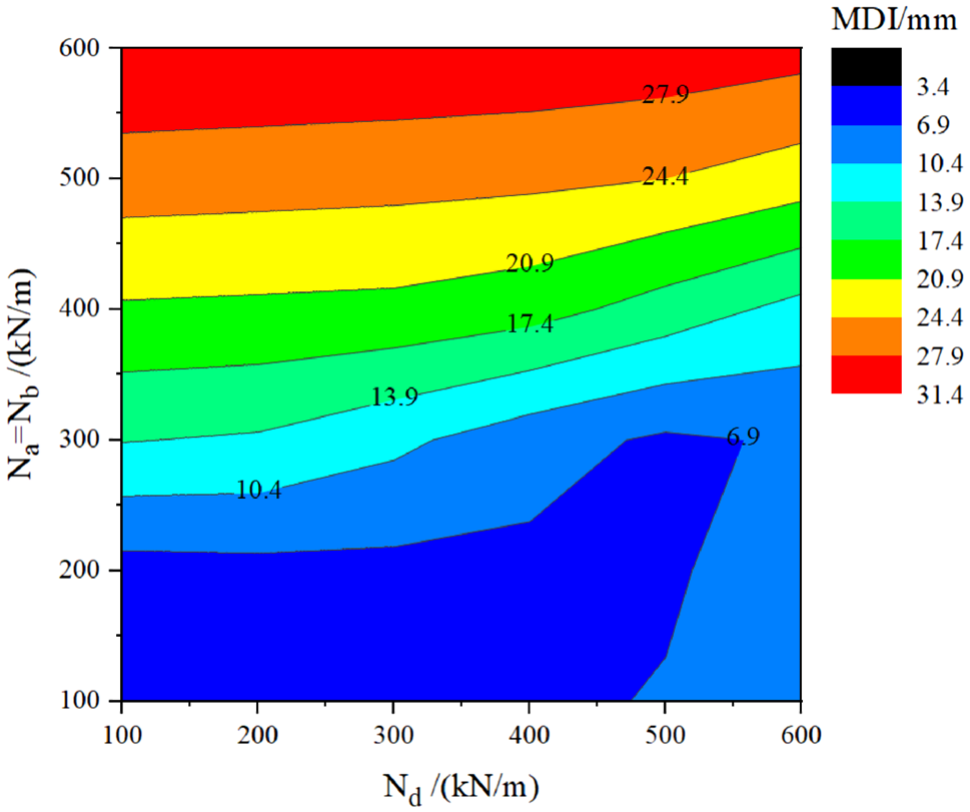
Contour chart of MDI with respect to the vertical forces (N_a_ = N_b_) and the transverse force N_d_ (N_c_ = 400 kN/m).

It is assumed that the axial force in the two vertical temporary linings varies simultaneously (N_a_ = N_b_); and that one of the axial forces in the transverse temporary lining N_c_ is set as 400 kN/m, while the other transverse force N_d_ varies independently. The calculation result is displayed in Table 7 and Fig. 16 under the following conditions: DMG is set as 200 MPa/m, and TPL is set as 400 mm.

**Table 7:** MDI location under different AFTLs in group C.

N_a_ = N_b_ =	N_c_ = 400 kN/m, N_d_ =			
	200 kN/m	300 kN/m	500 kN/m	600 kN/m
200 kN/m				
400 kN/m

As shown in Table 7, when N_a_ = N_b_ = 200 kN/m, N_c_ = 400 kN/m, and N_d_ = 200 kN/m, the MDI location is near the N_c_ action point. This is because the dominant force dominates the MDI and its location. In this example, N_c_ = 400 kN/m is the dominant force. Increasing N_d_ from 200 to 300 kN/m makes the MDI slightly increase, but the MDI location is still near the N_c_ action point. When further increasing N_d_ from 300 to 500 kN/m, the MDI increases, and the MDI location moves to near the N_d_ action point because of the same reason that the dominant force dominates the MDI and its location. When N_d_ increases from 500 to 600 kN/m, the MDI increase rate is more appreciable, and the MDI location gets closer to the N_d_ action point.

Moreover, the difference in MDI caused by increasing N_d_ from 200 to 300 kN/m is much smaller than that caused by increasing N_d_ from 500 to 600 kN/m. It implies that when N_d_ is smaller than N_c_, the MDI location is near the N_c_ action point, and N_d_ has little effect on the MDI. On the contrary, when N_d_ is more significant than N_c_, the MDI location is near the N_d_ action point, and N_d_ directly affects the MDI. On the other hand, when N_a_ = N_b_ = 400 kN/m, N_c_ = 400 kN/m, and N_d_ = 200 kN/m, the MDI is located at the invert. With N_d_ increasing from 200 to 600 kN/m, the MDI location is always at the invert, but the MDI decreases. This is because the dominant force dominates the MDI and its location and because the forces in vertical and transverse linings are suppressing each other.

Moreover, the difference in MDI caused by increasing N_d_ from 200 to 300 kN/m is much smaller than that caused by increasing N_d_ from 500 to 600 kN/m. It implies that when N_d_ is smaller than N_c_, N_c_ is dominant in the suppression effect on the MDI; Otherwise, N_d_ is dominant in comparison with Table 6 where N_c_ = N_d_, a sudden increase or decrease in the MDI is not observed in the current case where N_c_ ≠ N_d_.

Figure 16 presents the relationship among the MDI, the vertical forces (N_a_ = N_b_), and one of the transverse force N_d_ under condition of N_c_ fixing to 400 kN/m. When the vertical forces N_a_ and N_b_ are greater than 400 kN/m, varying N_d_ from 100 to 600 kN/m makes the MDI decrease, but the MDI decrease rate is lower than that in Fig. 15. When N_a_ = N_b_ = 250 kN/m, the MDI will decrease then increase with N_d_ varying from 100 to 600 kN/m. This is because of the change of the MDI location. When N_a_ and N_b_ are smaller than 200 kN/m, increasing N_d_ from 100 to 600 kN/m makes the MDI increase, since the MDI location is always at the sidewall.

### Countermeasure and tunneling method optimization

In this paper, displacement risks associated with dismantling the two conventional types of temporary linings have been evaluated using the bedded-beam model. From the results above, some countermeasures to control the risks can be proposed:

1. Replacing temporary linings with pre-tensioned anchor cable or bolt in order to avoid the adverse effects of forces in temporary linings during temporary lining dismantling stages.
2. Involving multi-layered preliminary linings to enhance the TPL.
3. Adopting grouting to improve the ground. From the sensitivity of these three factors, the first countermeasure would be efficient in controlling the risks in tunnel.

**Figure 17:**
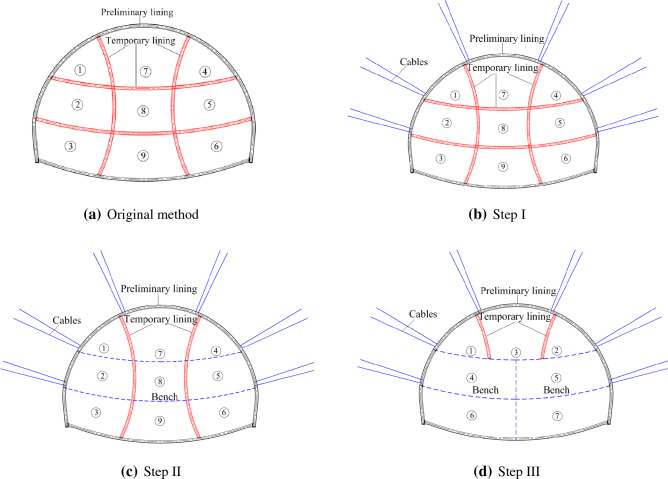
The optimization route about the tunneling method.

According to the risk-control countermeasures above, the tunneling method can be progressively optimized with the help of the pre-tensioned anchor cables in the following way (see Fig. 17):

1. Excavating tunnel in the sequence according to the original method shown in Fig. 17a; and replacing temporary linings with pre-tensioned anchor cables as shown in Fig. 17b. The preliminary lining as well as temporary lining is constructed immediately after every construction round. Before dismantling the temporary linings, installing pre-tensioned anchor cable to reduce the AFTL (see in Fig. 17b).
2. The role of transverse temporary lining would be replaced by that of pre-tensioned anchor cables, and thereafter, the temporary transverse linings can be removed after the installation of pre-tensioned anchor cables (in Fig. 17c).
3. The vertical temporary linings can be cut short, and the drifts would be adjusted correspondingly, as shown in Fig. 17d.

### Optimized tunneling method application

**Figure 18:**
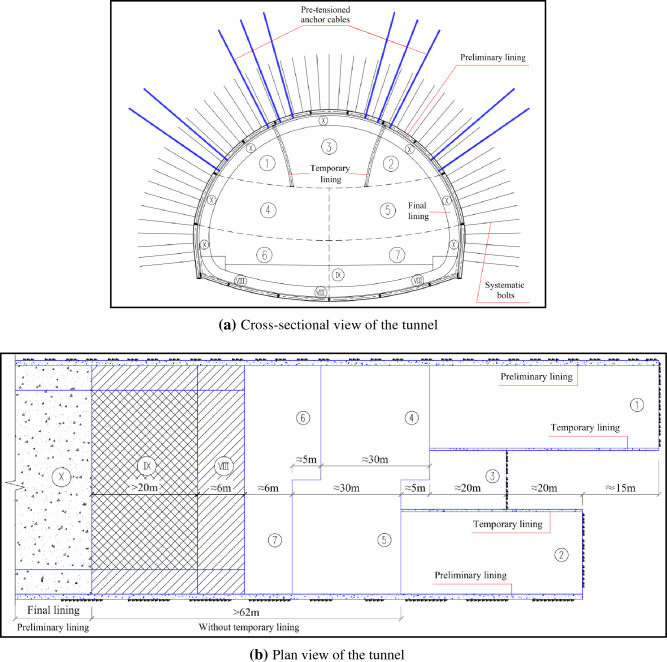
Final adopted tunneling method.

**Figure 19:**
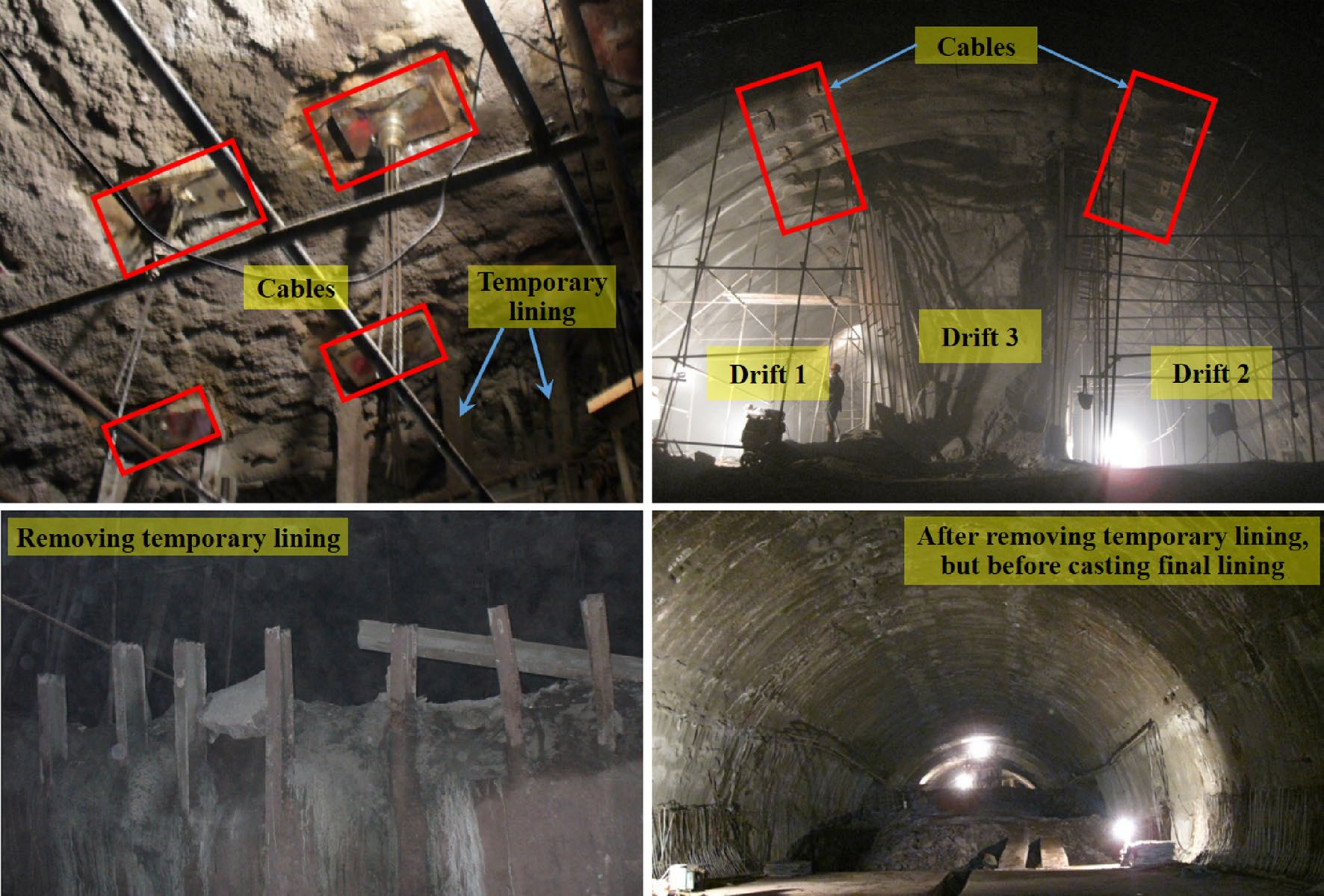
On-site photos of the temporary linings replaced by pre-tensioned anchor cables.

Through optimizations on-site, the final adopted tunneling method is presented in Fig. 18. The longitudinal and transverse spacing of the anchor cables are both 1.2 m. For each cable, the free length and bonding length are both 10 m; and the pre-tension force is initially set as 500 kN, later adjusted to 300 kN. The pre-tensioned anchor cables are vital to controlling the risk of dismantling temporary linings. From the plan view of the tunneling method in Fig. 18b, a long section (more than 62 m) can be constructed without temporary linings between the upper bench and final lining. In this way, project construction period and costs can be greatly reduced by removing the limited working space restraints resulting from the installation of temporary linings, and hence by accommodating the functions of heavy duty machines/equipment in this long section. Figure 19 presents the on-site photos about adopting the pre-tensioned anchor cable to replace temporary linings. There is no sudden increase in monitoring data during the period of dismantling the temporary linings.

## Conclusions

1. Based on the three influence factors, several countermeasures to decrease the risks of dismantling the temporary linings are put forward. By using the pre-tensioned anchor cables, most of the temporary linings in TM-2 are removed, and the axial force in the remained temporary lining is transferred to cables before removing the temporary linings. The optimized tunneling method is verified on site and shown to effectively control the risk of removing the temporary linings.
2. TM-1 always induces invert uplift, whereas TM-2 mainly brings about invert uplift or sidewall bulging depending on which linings, transverse or vertical, are dominant in terms of axial force values. When the axial forces in the vertical temporary linings are dominant, the deformation increment about preliminary linings caused by removing the temporary lining is mainly manifested as the uplift at the invert. If the axial forces in the temporary transverse linings are significantly greater than that in the temporary vertical lining, the MDI location about the preliminary linings will occur at the sidewall.
3. In TM-1 (double vertical temporary lining), if the axial force in double vertical temporary linings simultaneously increases (N_a_ = N_b_), the relationship between the MDI and the axial forces is linear. Otherwise (N_a_ ≠ N_b_), the MDI will significantly increase compared with the case of N_a_ = N_b_. In TM-2 (double vertical and double transverse temporary linings), the MDI is also linearly related to the *AFTL* when N_a_, N_b_, N_c,_ and N_d_ simultaneously increase (i.e. N_a_ = N_b_ = N_c_ = N_d_). The axial force in the transverse temporary lining (N_c_ and N_d_) suppresses the MDI when the MDI is located at the invert, while N_c_ and N_d_ enhance the MDI after the MDI location moves to the sidewall.
4. The TPL affects the MDI in the way that can be expressed using a power function. MDI reduces with the TPL, while the trend weakens with the increase of TPL. Similarly, MDI shows a power function relationship with DMG.

## Data Availability

Data will be available by the corresponding author on reasonable request.
